# Benchmark Study of Epoxy Coatings with Selection of Bio-Based Phenalkamine versus Fossil-Based Amine Crosslinkers

**DOI:** 10.3390/molecules28114259

**Published:** 2023-05-23

**Authors:** Pieter Samyn, Joey Bosmans, Patrick Cosemans

**Affiliations:** SIRRIS, Department of Innovations in Circular Economy and Renewable Materials, 3001 Leuven, Belgiumpatrick.cosemans@sirris.be (P.C.)

**Keywords:** epoxy, bio-based, phenalkamine, crosslinking, coating, mechanical properties

## Abstract

The phenalkamines (PK) derived from cardanol oil can be used as a bio-based crosslinker for epoxy coatings as an alternative for traditional fossil amines (FA). First, the reaction kinetics of an epoxy resin with four PK and FA crosslinkers are compared by differential scanning calorimetry, illustrating a fast reaction rate and higher conversion of PK at room temperature in parallel with a moderate exothermal reaction. Second, the performance of coatings with various concentrations of PK and PK/FA ratios indicates good mixing compatibility between crosslinkers resulting in higher hardness, scratch resistance, hydrophobicity, and abrasive wear resistance of coatings with PK. The superior performance is confirmed over a broad range of resin/crosslinker ratios, facilitating the processing with viscosity profiles depending on the PK type. Although fossil- and bio-based crosslinkers have different chemical structures, the unique linear relationships between intrinsic mechanical properties (i.e., ductility and impact resistance) and coating performance indicate that the degree of crosslinking is a primary parameter controlling coating performance, where PK simultaneously provides high hardness and ductility. In conclusion, the optimization of the processing range for bio-based PK as a crosslinker for epoxy coatings delivers suitable processing conditions and superior mechanical performance compared to traditional amine crosslinkers.

## 1. Introduction

In recent years, the incorporation of bio-based building blocks in protective polymer coatings has been stepwise implemented in industrial cases in parallel with the need for reducing environmental impact and scarcity in fossil-based resources. The epoxies are preferred as solvent-free resin in composites, adhesives or coatings on concrete, stone, or wood because of mechanical durability (high hardness and abrasion resistance) [1,2], fungal decay resistance [3], corrosion protection, and chemical resistance [4]. It is mainly applied for indoor use (e.g., flooring and furniture) due to its sensitivity to ultraviolet degradation and lower scratch resistance compared to polyurethane coatings [5]. The mechanical and thermal properties of the thermoset coating can be tuned depending on the selection of molecular structures for the epoxy resin and crosslinkers, which result in the formation of a densely crosslinked polymer network [6,7]. The crosslinking reaction involves the opening of the epoxide ring or oxirane group in the presence of a crosslinking agent (or so-called curing agent or hardener), such as (poly)amines, acids, anhydrides, polyols (phenol and alcohols), or thiols [8]. In general, the mechanical scratch resistance of epoxy coatings increases with higher crosslink density and increasing glass transition temperature [9]. However, the brittleness and poor impact resistance of epoxy may limit its use in tribological applications, whereas the introduction of reinforcing agents, such as glass fibers [10], natural fibers [11], hybrid fibers [12], or mineral fillers [13], were considered to improve the wear resistance. Several toughening mechanisms for epoxy resins were observed in nanocomposites [14] or elastomer-modified epoxy resins [15], as reviewed before [16]. Eventual preprocessing of the epoxy under high pressure and shear rates [17] or controlled thermal curing [18] was able to improve the fracture toughness as a result of an increase in polymer chain length together with a reduction in crosslinking density.

The recent advances in bio-based epoxy resins and crosslinkers indicated a fast growth in synthesis from modified plant oils, saccharides, natural polyphenols, terpenes, rosin, natural rubber, and lignin [19,20,21,22]. The bio-based aromatic compounds for replacement of epoxy prepolymers were synthesized [23] in parallel with bio-based aromatic crosslinkers [24], which are expected to provide better properties for high-performance coatings compared to aliphatic or cycloaliphatic fossil-based amines (FA). The bio-based phenalkamine (PK) crosslinkers for epoxy are synthesized from cardanol oil, which is present in the distillate from cashew nutshell liquid (CNSL), including 1 to 9% cardanol, 74 to 77% anacardic acid, and 15 to 20% cardol and 2-methyl cardol [25], respectively. The Mannich reaction of cardanol in the presence of formaldehyde and aliphatic diamines, such as ethylenediamine, diethylenetriamine, or triethylenetetramine, results in the substitution of a polymer chain with amine functionalities onto the aromatic cardanol structure [26]. Alternative synthesis routes for amine-functionalized cardanol were developed without the drawback of using hazardous products, such as formaldehyde or toxic amines; therefore, a new aromatic amine was prepared by thiolene coupling of cardanol with cysteamine hydrochloride and similar performance compared to commercial PK was demonstrated [27]. The aromatic phenol group within PK results in a crosslinked epoxy network with high rigidity, thermal stability, and chemical resistance, while the presence of a C15 aliphatic sidechains in meta position provides flexibility, hydrophobicity, viscosity control, and wettability [28]. The residual hydroxyl groups on the phenol structure aid in superior adhesion to various substrates [29] and fast curing properties at ambient temperature [30]. The reactivity of PK for epoxy crosslinking was experimentally verified in combination with different types of amines, where an improvement in the toughness of the epoxy resin was noticed compared to traditional diethylenetriamine [31]. Epoxy curing is characterized by an exotherm reaction, for which the activation energy depends on the steric hindrance of the PK and is a diffusion-controlled process of the epoxy and amine molecules at the initial state [32,33]. The synthesis of PK with different polyamines revealed that they performed as well as a crosslinker for epoxy resins providing mechanical, optical, thermal, and anticorrosive properties [34]. The kinetics and mechanism of the crosslinking reaction between amines and epoxy were reviewed [35], confirming the importance of hydrogen bonding in the transition state of a termolecular mechanism. The hardness, flexibility, tensile, and flexural strength could be tuned depending on the chain length and reaction conditions of the PK [36]: however, it was observed that the mechanical strength could decrease with an increase in bio element as the crosslinking ability of the epoxy was reduced. Therefore, the PK with higher molecular weight was synthesized by varying the molar concentrations of the reaction ingredients, resulting in faster surface drying and better mechanical properties [37]. In general, the crosslinking of epoxy composites with PK is promising as their visco-elastic properties can be controlled without presenting brittle failure [38]. Additionally, the water and moisture resistance of epoxy composites could be reduced in the presence of PK depending on the selection of suitable curing conditions [39], which make them favorable for use in maritime environments. The better impact strength and elongation of crosslinked epoxy composites with PK could result in better wear resistance under dry adhesive sliding [40].

In this study, the crosslinking of epoxy coatings with a selection of traditional fossil-based amines and bio-based PK is systematically investigated for a selection of commercially available grades, in order to identify favorable application conditions with improved wear resistance and ductility. The aim is to compare performance for a broad range of crosslinkers on the crosslinking kinetics of coating properties, including the effects of resin/crosslinker ratio and curing conditions. Additional insight into the mechanical properties and failure mechanisms are obtained through the evaluation of wear mechanisms in parallel with the degree of crosslinking. The latter is related to the intrinsic mechanical properties, such as ductility and impact resistance. In addition to the available literature cited above, mainly focusing on the chemical synthesis and crosslinking mechanisms, the novelty of the present study lies in a better understanding of the relationship between intrinsic mechanical properties and coating performance by systematically replacing fossil-based amines with PK. The results support the use of a bio-based crosslinker for protective epoxy coatings on wood (e.g., flooring and furniture) chosen as a model system, which may provide better mechanical and physicochemical properties than epoxy coatings with a fossil-based crosslinker after optimization of the processing conditions. 

## 2. Results

### 2.1. Analysis of Processing Conditions: Crosslinking and Viscosity

**Figure 1:**
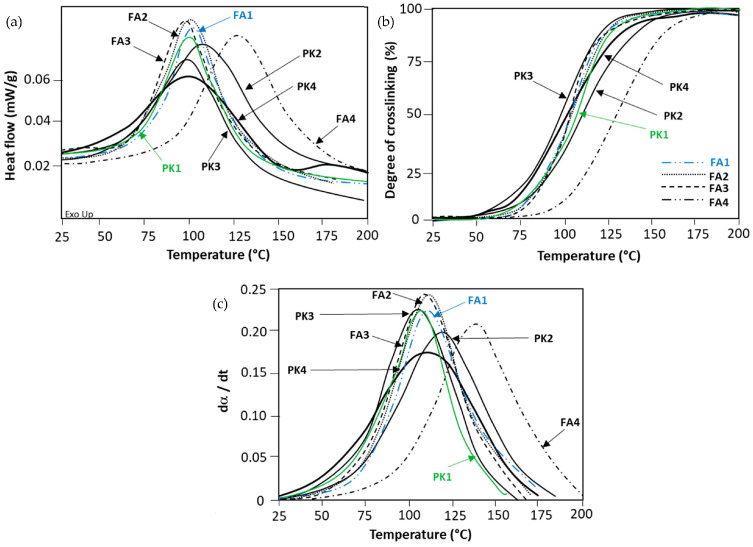
Crosslinking reaction between DGEBA and FA or PK as measured by non-isothermal DSC analysis on liquid coating samples: (**a**) heat flow curves, (**b**) degree of crosslinking or conversion, (**c**) conversion rate.

After formulation of coatings with DGEBA epoxy resin and fossil amines (FA1 to FA4) or bio-based phenalkamines (PK1 to PK4) as crosslinkers added in stoichiometric ratio, the thermal curing kinetics were evaluated from non-isothermal differential scanning calorimetry (DSC) on liquid coating samples. The crosslinking reaction between the epoxy ring and the amine group is characterized by an exothermal peak in the DSC thermogram (Figure 1a), from which the degree of crosslinking or conversion can be calculated (Figure 1b) together with the conversion rate dα/dt (Figure 1c).

The crosslinking mechanisms and kinetics of epoxy resins have been frequently studied through evaluation of the heat release under isothermal [41] or non-isothermal DSC analysis [42]. The crosslinking process is mainly characterized by the starting temperature, broadness of the temperature interval, and the reaction speed that is equivalent to the conversion rate. While scanning over the full temperature interval, reactivity at room temperature and maximum reaction temperatures can be determined. The characteristics of the crosslinking reaction depend on the type of crosslinker while using the same epoxy resin, with some overlap in optimum temperature ranges for FA and PK. In order to balance with the final mechanical properties, however, often mixtures of amine crosslinkers containing linear, branched, and aromatic amines are applied. For FA, a broad temperature range and slow crosslinking kinetics was observed for FA4 because of the slow cure of polyetheramine owing to its chemical structure. The crosslinking reaction is better controlled and optimized with a more complex molecular mixture of amines in FA3. The selection of simple aliphatic amines (FA2) or cyclic amines (FA1) provides comparable crosslinking characteristics that are mainly determined by the presence of reactive sites in the molecular structure: depending on the amine composition, high reactivity is due to the portion of present reactive hydrogen groups in parallel with low molecular weight. For PK, the crosslinking kinetics are in a similar range to selected FA, with minor differences indicating a faster crosslinking at temperatures below 50 °C (room temperature). The latter is owing to the presence of phenolic groups and reactive hydroxyl groups in PK. Depending on the composition of PK, however, the curing speed can be controlled through blending with amines [43], as illustrated by the presence of higher concentrations of m-phenylene bis(methylamine) in PK3 and PK4 (faster curing) relative to the presence of lower concentrations of m-phenylene bis(methylamine) in PK2 (slower curing). Additionally, the exothermal enthalpy for crosslinking of epoxy with PK is lower (see peak height, Figure 1a), and the reaction evolves slower (see conversion slope, Figure 1b) as an advantage for better process control compared to FA. Indeed, the crosslinking of epoxy with amines is an autocatalytic process where the released heat is an accelerator for further processing [44]. The full conversion for selected crosslinkers was obtained in the appropriate temperature region with a maximum reaction rate of around 100 °C. The S-shape curve with a slowing down in conversion rates as a function of higher conversion degrees is expected as functional groups were reacted. While comparing the reactivity of PK2, PK3, and PK4, the amine with higher AHEW is expected to have the lowest maximum reaction rate dα/dt. From the overview of chosen crosslinkers, the fossil-based FA1 (see blue curve) and bio-based PK1 (see green curve) provide comparable reaction kinetics and are selected for further coating evaluations. 

**Figure 2:**
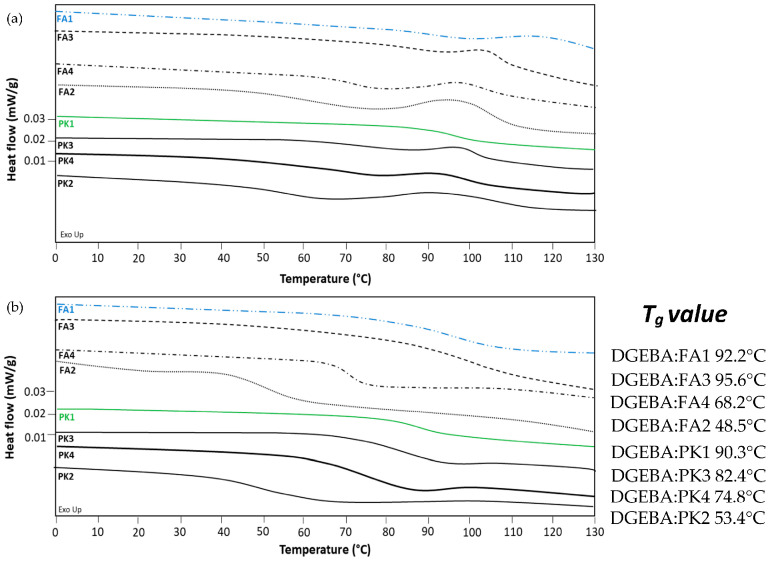
DSC analysis of crosslinked DGEBA coatings with FA1 to FA4 and PK1 to PK4, (**a**) first heating scan, (**b**) second heating scan.

Thermal characteristics of crosslinked epoxy coatings were determined by DSC during the first heating (Figure 2a) and second heating (Figure 2b). Aside from the previous evaluation of exothermal heat release, the crosslinking of epoxy can also be evaluated by monitoring the glass transition temperature *T_g_* [45]. The first heating indicates a weak *T_g_* and residual exothermal peak at around 100 °C. The latter is most expressed for fossil-based FA, indicating the presence of residual non-crosslinked material in parallel with a more difficult crosslinking at low temperatures. The residual exothermal peak is present for some PK crosslinkers in lower intensity, in parallel with the previously noticed reaction kinetics for PK that present a faster crosslinking at room temperature compared to FA. The successful crosslinking in the second heating is expressed as a clear *T_g_* and absence of further exothermal reaction. The *T_g_* relates to the structural composition of the amine crosslinker, as the highest *T_g_* is observed for the epoxy coatings with cyclic amines (FA1) or branched amines (FA3), and lower *T_g_* is observed for short aliphatic amines (FA2) or polyetheramines (FA4). The *T_g_* is thus in parallel with the expected molecular mobility and flexibility of the amine linkages. Additionally, depending on the composition of PK, the mixtures with low-molecular weight amines (PK2 to PK4) have a lower *T_g_* than pure phenalkamine (PK1). Depending on the degree of crosslinking, the formation of a denser polymer network in the presence of the reactive di-amines with higher functionality decreases the molecular mobility and provides the higher *T_g_*. The presence of the long alkyl chains in PK increases the flexibility of the polymer chains and, hence, decreases the glass transition temperature, while the somewhat higher *T_g_* for PK 3 is in parallel with the higher reactivity of this crosslinker as demonstrated before, attributed to the presence of m-phenylene bis(methylamine) in higher concentrations than other PK. For the selected FA1 (see blue curve) and bio-based PK1 (see green curve), similar ranges in *T_g_* are obtained, and the ones with relatively high *T_g_* values are selected for further mechanical coating evaluation. 

**Figure 3:**
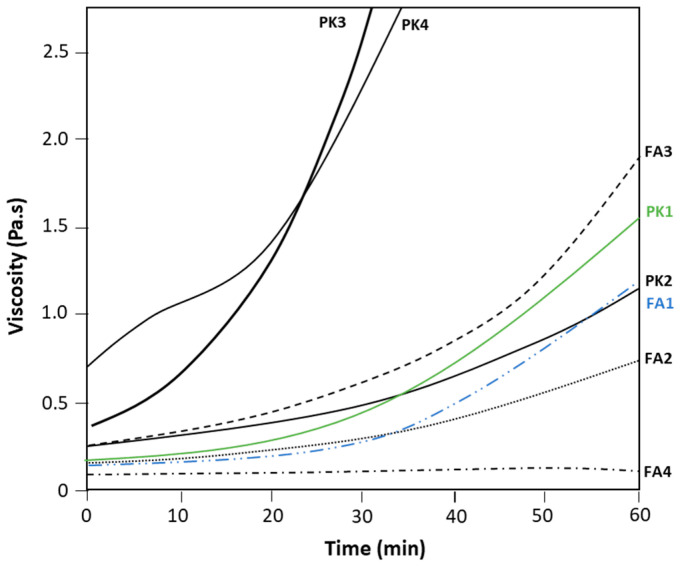
Viscosity measurements over time at a constant rotational speed of DGEBA coatings with FA1 to FA4 and PK1 to PK4 crosslinkers.

The mixing of epoxy resin with respective crosslinkers was also followed by viscosity measurements over time at room temperature (Figure 3). The increase in viscosity over one hour of mixing can be associated with the onset of the crosslinking reaction and relates to the reactivity of the amine crosslinker. This method has, therefore, also been used before to monitor the reaction rate and operational pot-life of epoxy [46]. The initial viscosities of epoxy coatings with fossil or bio-based amines are within similar ranges, as the specific commercial grades of PK with low-viscosity were selected. For FA, the viscosity of simple aliphatic or cyclic amines (FA1, FA2) is relatively low, and they provide appropriate reactivity within about 30 min. The reactivity of branched amines with higher functionality (FA3) is evidently higher and results in a faster increase in viscosity upon reaction. The long-chain polyetheramine (FA4) provides the lowest viscosity in parallel with a low reactivity at room temperature, according to previous DSC analysis. For PK, the high reactivity at room temperature is evidenced by a fast increase in viscosity after a short mixing time. The sharp viscosity increases due to high reactivity at room temperature, in particular for PK3 and PK4, was also noticed in fast conversion during DSC analysis, while the conversion rate for PK4 was somewhat slower compared to PK3. The slower increase in viscosity for PK2 also could be expected in parallel with the slower conversion rates as observed in DSC measurements, while PK1 presents a somewhat higher reactivity at room temperature relative to PK2. The viscosity profiles for FA1 (see blue curve) and PK1 (see green curve) are very similar, and these grades were further selected for mechanical coating evaluations.

### 2.2. Preselection of Different Crosslinkers: Effect on Coating Properties

**Figure 4:**
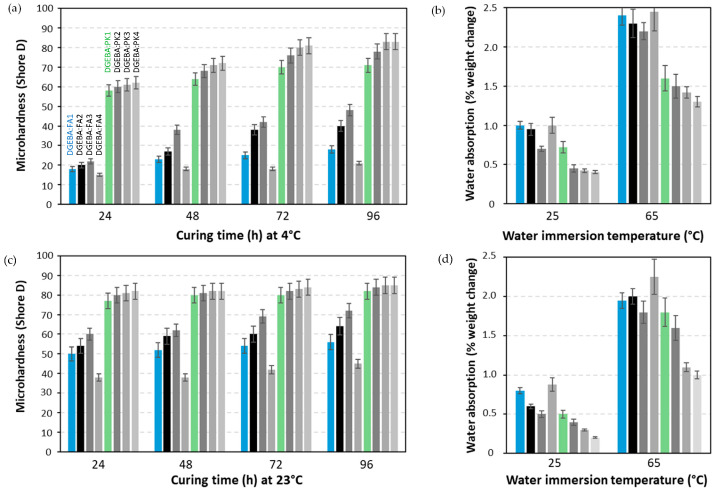
Benchmarking of DGEBA epoxy coatings with FA1 to FA4 and PK1 to PK4 crosslinkers as wood coatings under different curing temperatures and times, (**a**) microhardness after curing at 4 °C for different times, (**b**) water absorption for 2 h at 25 or 65 °C, after curing at 4 °C for 96 h, (**c**) microhardness after curing at 23 °C for different times, (**d**) water absorption for 2 h at 25 or 65 °C, after curing at 23 °C for 96 h. Every bar represents different coating composition at stoichiometry, as indicated.

The effects of crosslinking conditions on the properties of epoxy coatings were evaluated for DGEBA with FA1 to FA4 and PK1 to PK4 crosslinkers that were mixed in a stoichiometric ratio. The coating formulations were subsequently applied on wood substrates and cured at low temperature (4 °C) and room temperature (23 °C) for 24 to 96 h. The variations in microhardness and water absorption of the coatings were measured for the different coating compositions and reaction conditions (Figure 4). For FA, the weak crosslinking reaction at room temperature, as demonstrated in the previous DSC, results in relatively low microhardness and high sensitivity to water absorption. In particular, the very slow reaction rate of epoxy coatings with FA4 corresponds to the weakest mechanical properties, while the more complex amines with branched structures and higher amine functionality (FA3) provide better mechanical properties compared to the linear aliphatic amines (FA2). The fast reaction of epoxy coatings with PK is observed as the microhardness is established relatively fast, even for curing at low temperatures. In parallel, the water absorption of coatings with PK is lower than coatings with FA crosslinkers. The improvement in mechanical properties and reduced water absorption can be related to the high crosslinking densities of the coatings with PK, as demonstrated in the previous paragraph. From these observations, it can be concluded that PK is favorable for fast crosslinking and the introduction of good mechanical properties and water resistance to the coating even after short reaction times and at low temperatures, without the need for additional thermal treatment. 

### 2.3. Crosslinking Conditions of Epoxy Coating: Effect on Mechanical Properties

**Figure 5:**
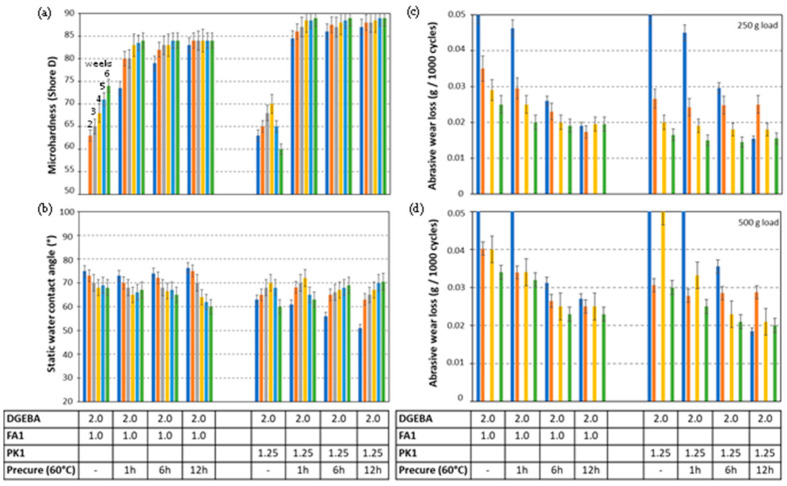
Influence of curing conditions on mechanical properties of DGEBA:FA1 and DGEBA:PK1 coatings under stoichiometric mixing ratio, with different thermal precuring times of 0, 1, 6, and 12 h at 60 °C and supplementary curing at room temperature for 1 week (dark blue bar), 2 weeks (orange bar), 3 weeks (grey bar), 4 weeks (yellow bar), 5 weeks (light blue bar), and 6 weeks (green bar), (**a**) microhardness, (**b**) static water contact angle, (**c**) abrasive wear under low load, (**d**) abrasive wear under high load. Every bar represents longer curing time at room temperature as indicated, the numbers in the table mixing ratio (g/g).

The relationships between crosslinking conditions and the mechanical performance of epoxy coatings with FA1 and PK1 are further analyzed in detail. In the following comparative studies, both crosslinkers are selected based on the similar *T_g_* values of the cured coatings (see Figure 2b). After stoichiometric mixing DGEBA:FA1 (2:1) and DGEBA:PK1 (2:1.25), the different thermal precuring times of 0, 1, 6, and 12 h at 60 °C were applied followed by measurements of microhardness, static water contact angle and abrasive wear loss after additional curing for 1, 2, 3, 4, 5, and 6 weeks under laboratory conditions (50% RH, 23 °C) (Figure 5). The microhardness of epoxy coatings with FA1 progressively increases with time and temperature, while thermal precuring signifies an important factor in improving the microhardness (Figure 5a). The epoxy coatings with PK1 attain higher hardness after.

Mild thermal precuring (shorter times) and also after short additional curing times under laboratory conditions. From this, the benefits of fast crosslinking of epoxy coatings with PK are illustrated. However, more instabilities are observed for PK1 coatings without thermal precuring, likely due to the higher sensitivity for structural reorganizations in the coating after deposition. In particular, the decrease in hardness after 4 to 5 weeks can likely be attributed to migration effects in the coating. Besides the evidence for fast curing of the coating bulk and favorable bulk properties of the coating, the curing conditions might have a stronger influence on the surface properties. The static water contact angles confirm this trend because a reduction in static water contact angles is expected for the more crosslinked coatings, as observed for the coatings with FA1 (Figure 5b). However, the opposite trends with increasing water contact angles over time are often observed for PK1 coatings as an indication of migration effects of non-crosslinked moieties towards the coating surface. The presence of small reactive species has high mobility through the coating, and their accumulation at the coating surface may cause different surface effects compared to the bulk. The abrasive wear was evaluated for some selected coatings under low loads (Figure 5c) and high loads (Figure 5d), while further details on measurements of abrasive wear loss as a function of the number of abrasive cycles are given in Supplementary Information Figure S1. A progressive reduction in abrasive wear with longer curing times is observed, whereas the abrasive wear loss of PK1 coatings is lower than FA1 coatings, in parallel with the higher hardness of PK1 coatings.

**Figure 6:**
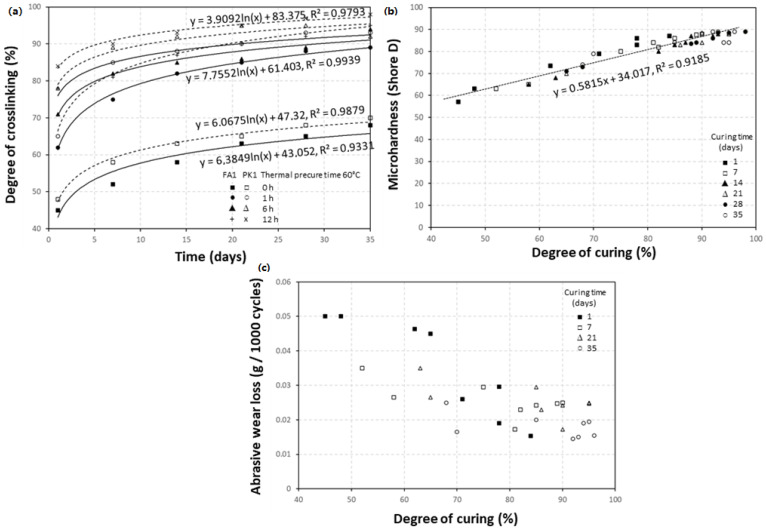
Relationships between mechanical performance and crosslinking of DGEBA:FA1 and DGEBA:PK1 under different crosslinking conditions, including precuring at 60 °C and additional curing under laboratory conditions for several weeks, (**a**) degree of crosslinking over time with some exemplary regression curves, (**b**) microhardness as function of crosslinking including both FA1- and PK1-epoxy coatings, (**c**) abrasive wear loss as function of crosslinking including both FA1- and PK1-epoxy coatings.

The changes in mechanical properties of the epoxy coatings can mainly be related to the degree of crosslinking. The DGEBA:FA1 (1:1) and DGEBA:PK1 (1:1.25) coatings under different conditions of precuring for 0, 1, 6, and 12 h at 60 °C and subsequent curing times over 1, 2, 3, 4, 5, and 6 weeks under laboratory conditions (50% RH, 23 °C) were evaluated. As a summary, the explicit relationships between the degree of crosslinking, microhardness, and abrasive wear loss are illustrated in Figure 6 for the FA1 and PK1 coatings under different curing conditions. The thermal precuring evidently increases the initial crosslinking in the coating, which further gradually increases during subsequent curing for several weeks under laboratory conditions. The respective trendlines could be fit to a logarithmic function (some examples of the equations are inserted in Figure 6a), with statistical significance value R2 > 0.93 (for weakly cured samples) to R2 > 0.98 (for strongly cured samples). The microhardness of coatings evidently relates to the degree of crosslinking (Figure 6b) with statistical significance value R2 = 0.92, as the stronger crosslinking of the epoxy coating corresponds to more rigid bulk properties and better mechanical resistance. As a universal measure of hardness versus degree of crosslinking and similar crosslinking mechanisms for the used amine-based crosslinkers, the same linear trend is covered for both FA1 and PK1 crosslinkers. The abrasive wear loss of the coatings decreases with increasing hardness and/or increasing degree of crosslinking (Figure 6c); however, a higher spread is observed in the relationships for weakly crosslinked coatings (e.g., coatings without thermal precure). In the latter case, transient wear mechanisms are related to a combination of both bulk and surface properties that are not only covered by mechanical hardness. The residual moieties in weakly crosslinked coatings may highly affect the surface properties and contribute to a variation in wear.

### 2.4. Composition of Epoxy Coating: Effect on Mechanical Properties

The effect of different mixing ratios of resin versus crosslinker on the mechanical properties of the coatings was evaluated in order to have an indication of sensitivity in coating composition on coating performance. In the first testing series, the DGEBA:FA1 coating compositions were varied in weight ratios of resin versus FA1 (2:1.5 to 2:3.5, stoichiometric 2:1), and DGEBA:PK1 coating compositions were varied in weight ratios of resin versus PK1 (2:0.85 to 2:1.65, stoichiometric 2:1.25). Subsequently, the compatibility between fossil-based and bio-based crosslinkers was evaluated by adding them in different weight ratios of FA1:PK1. Indeed, the crosslinking for FA and PK amines should follow the same mechanism regardless of their origin but with different kinetics, as illustrated before, as the reactivity of the amine depends on its configuration and nucleophile nature. According to the best processing conditions resulting in highly crosslinked coatings based on the above test results, the respective coatings were thermally precured for 12 h at 60 °C followed by additional curing for 6 weeks under laboratory conditions (50% RH, 23 °C).

**Figure 7:**
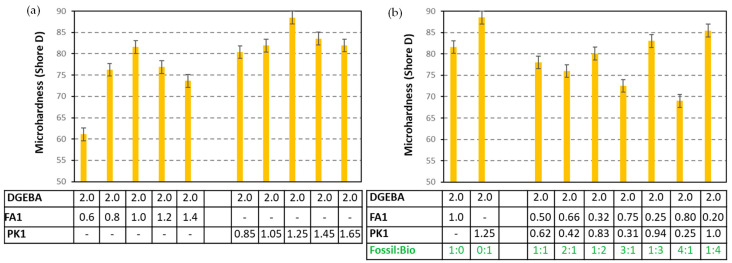
Microhardness measurements of DGEBA:FA1 and DGEBA:PK1 coatings with different mixing ratios, (**a**) mixing ratio of crosslinker versus epoxy resin, (**b**) mixing ratio of fossil-based FA1 versus bio-based PK1 crosslinker, numbers in the table are mixing ratio (g/g).

The microhardness of coatings is obviously highest for compositions under stoichiometric mixing ratios (Figure 7a), while the bio-based PK1 provides higher hardness compared to the fossil-based FA1. The higher hardness for PK-epoxy coatings is in line with the higher degree of crosslinking for PK-epoxy than for FA-epoxy coatings, as determined in the previous section. The high hardness, tensile, and flexural strength of PK-epoxy compared to conventional FA-epoxies was also confirmed in other studies, depending on the selection of the phenalkamine [36], where the crosslinking density of the PK-epoxy coatings was higher compared to FA-epoxy coatings. Moreover, the variations in microhardness for coating compositions with different mixing ratios are more pronounced for the fossil-based FA1 compared to the bio-based PK1. Therefore, the latter crosslinking with phenalkamine is less sensitive to the exact mixing ratio of crosslinker versus epoxy resin because it is also more reactive in the crosslinking reaction at room temperature, as confirmed by previous DSC measurements. The good reactivity of cardanol-derived PK and fast cure at room temperature can be attributed to the long aliphatic side chains that are accessible for crosslinking and the reactive hydroxyl group near the aromatic ring structure. For a relatively broad range in the mixing ratio of bio-based PK1, the microhardness of the coatings only varies in a small range, and its value remains above the hardness of the fossil-based FA1. The latter may be favorable for the processing of a bio-based epoxy composition and simplify the mixing conditions. 

While mixing the bio-based PK1 and fossil-based FA1 crosslinkers in different ratios relative to each other in comparison with a given amount of epoxy resin (Figure 7b), the progressive increase in bio-based PK1 content steadily improves the microhardness of the coating in parallel with the degree of crosslinking. Although the long aliphatic chain lengths in the PK-structure would allow for higher coating flexibility, it seems that mainly the formation of a more densely crosslinked polymer network and high reactivity of PK-epoxy is dominating. In parallel, a linear increase in microhardness is observed with increasing content of the bio-based PK1 (see Supplementary Information Figure S2a). The experimental trend indicates that both fossil-based and bio-based crosslinkers can be combined to tune to final coating properties. 

**Figure 8:**
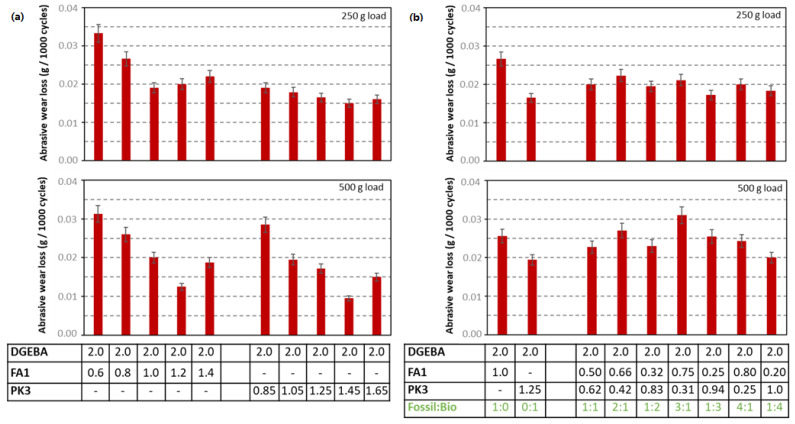
Abrasive wear loss under low load (top) and high load (bottom) of DGEBA:FA1 and DGEBA:PK1 coatings with different mixing ratios, (**a**) mixing ratio of crosslinker versus epoxy resin, (**b**) mixing ratio of fossil-based FA1 versus bio-based PK1 crosslinker, numbers in table are mixing ratio (g/g).

For similar variations in mixing ratios of crosslinker versus resin as above, the abrasive wear properties of the coatings are determined on a Taber tester under low load and high load, including coatings with FA1 or PK1 under different concentrations relative to the epoxy resin (Figure 8a), or mixtures between FA1 and PK1 in different ratios (Figure 8b). The actual measurements of wear loss as a function of the number of abrasive cycles are presented in Supplementary Information Figure S3. The lower wear for coatings with bio-based crosslinkers and linear trends over the full testing period is confirmed from the graphs under low loads (Figure S2a). A different wear behavior is observed under high loads (Figure S2b), where the wear for selected FA-epoxy coatings sharply increases after a certain number of cycles while the wear for selected PK-epoxy coatings rather flattens and stabilizes after a longer testing time. The nonlinearity in abrasive wear with load conditions indicates different wear mechanisms, where the wear stabilization of bio-based coatings is supported by their high hardness. Although there might be slight variations in the total wear depending on the applied load due to the actual governing wear mechanism, some general tendencies can be observed in parallel with the previous hardness measurements. The abrasive wear for coatings with bio-based crosslinker becomes lower than coatings with fossil-based crosslinker, and lowest wear rates are observed under stoichiometric mixing ratios in parallel with the highest hardness under these mixing conditions (Figure 8a). This is also true for the lower wear as the bio-based PK1 is progressively mixed with the fossil-based FA1 (Figure 8b). At higher loads, there is some statistical spread on the testing results, but a slight overdosing of the amine crosslinker can be beneficial for obtaining the lowest wear. In particular, the combination with plastic deformation for coatings with some lower hardness may provide lower wear rates. In abrasive wear conditions, it is very important to study the hardness of the organic coatings [47]. The latter is obviously related to the degree of crosslinking, as presented in previous paragraphs. The thermoset epoxies with high hardness are typically known for their intrinsic brittleness, while improvements in ductility can be realized with additives [48] or after yielding under plastic deformation and depending on the degree of crosslinking [49]. However, the hardness is not the sole bulk property that may determine the wear properties but also lubricating surface properties may become favored in the presence of an amount of free amine moieties, which was also discussed above in relation to the crosslinking and eventual migration effects. The wear properties are further documented by additional analysis of the wear tracks in the following paragraphs.

**Figure 9:**
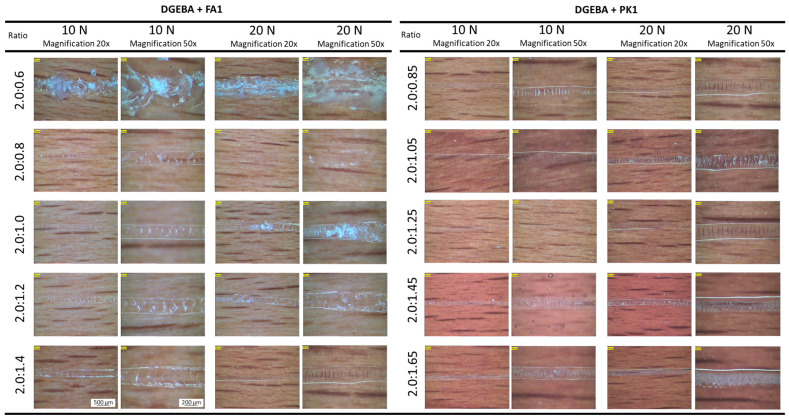
Scratch resistance of DGEBA:FA1 and DGEBA:PK1 coatings with different mixing ratios under 10 N and 20 N load, observed through optical microscopy at 20× and 50× magnifications (same scale bar as bottom left applies to all micrographs).

The results of scratch testing on epoxy coatings under 10 N and 20 N normal loads are presented through optical microscopy of the scratch under 20× and 50× magnification (Figure 9). More details for the scratching tests on coatings with various ratios of fossil versus bio-based crosslinkers are given in Supplementary Information Figure S4. It is clear that the best scratch resistance is obtained under 1:1 stoichiometric mixing condition in parallel with the highest microhardness values of the coatings. In parallel, the scratch resistance for the coatings with PK1 is superior to FA1, also in parallel with the higher hardness of the bio-based coating types. There is no surface damage to the bio-based coatings under low-load scratching. The differences in scratch mechanisms between coatings with fossil-based and bio-based crosslinkers are observed under the highest load conditions, where the fossil-based coatings present severe breakthrough of the coating. According to the literature, both the tensile strength and compressive yield stress determine the resistance against damage formation during scratching [50], which is influenced by the crosslinking density. The fully crosslinked epoxies are brittle, with poor resistance to crack initiation and growth [51]. The higher crosslinking of the epoxy coatings due to processing conditions or additives is known to enhance the scratch resistance, while hardness and scratch resistance are two mechanical properties that are related [52]. Previous studies also showed that the scratch resistance of epoxy coatings is to increase both with increasing degree of crosslinking and high *T_g_* [9], which can presently be confirmed in parallel with the calculated degree of crosslinking of the coatings that are higher for the bio-based epoxy coatings.

### 2.5. Wear Track Analysis of Epoxy Coatings 

**Figure 10:**
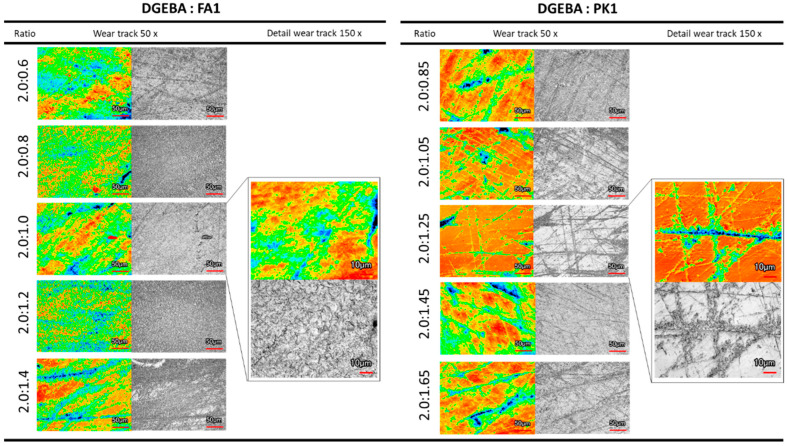
Evaluation of abrasive wear track of DGEBA:FA1 and DGEBA:PK1 coatings with different mixing ratios, observed through laser scanning microscopy at 50× and 150× magnifications.

The wear tracks for epoxy coatings are visualized by laser scanning microscopy, including optical images and three-dimensional topography. The results for epoxy coatings with different crosslinker ratios FA1 and PK1 (Figure 10) or variable conditions of thermal pre-curing (see Supplementary Information Figure S5) are illustrated. The differences in abrasive wear mechanisms between coatings with different crosslinkers are clearly observed with predominant wear of the bulk coating (fossil-based, FA1) or localized superficial wear scratches and formation of wear patches (bio-based, PK1). All material removal through wear remains localized within the coating and does not penetrate the wood substrate.

The low hardness of epoxy/FA1 coatings results in large plastic deformation in the wear track, as observed in the detailed analysis at high magnification. The local plastic deformation of fossil-based epoxies under dry abrasive wear has also been observed in ball-on-disc tribological tests for epoxy coatings on steel substrates [53], and severe material loss was attributed to wear debris peeling from the wear track [54]. Indeed, the existence of loose wear particles in the wear track may serve as third body abrasives. The poor wear resistance of native epoxy coatings is widely known due to its brittle nature, resulting in poor ductility and low fracture toughness [55]. This may be partially attributed to the internal stresses resulting from the crosslinking process [56]. Therefore, mechanisms that simultaneously enhance the hardness and ductility of epoxy matrixes are actively searched for in order to improve abrasive wear resistance and were currently demonstrated by incorporating a phenalkamine crosslinker.

The higher hardness of the bio-based coatings introduced better wear protection with only the formation of local surface scratches, in parallel with the low wear losses. However, the bio-based epoxy remains sensitive to the formation of some deeper grooves due to the brittleness of the hard polymer materials. It could be expected that the brittleness of bio-epoxy coatings with higher hardness compared to fossil-based epoxy coating is parallel with the higher degree of crosslinking (see Figure 6a) and could reduce the abrasive wear resistance. However, it has been stated before that softening effects and toughening of modified epoxy with rubbers increase the potential for internal energy dissipation in the materials, thereby reducing the abrasive wear [57]. Otherwise, the reduction in micro-cutting and micro-ploughing wear mechanisms for epoxy with PK crosslinker was previously attributed to the higher flexibility of the polymer chains and better adhesion between wear particles [40]. Alternatively, the smooth abrasive wear surfaces of bio-based epoxy coatings with PK may result from the intrinsic molecular structure, in agreement with the lower *T_g_* of the bio-based epoxy coating (see Figure 2b). The latter implies higher molecular mobility owing to the long aliphatic and flexible side chains in the PK, yielding possibility for better self-lubricating properties due to molecular mobility and orientation of the long polymer molecules near the sliding surface. For the same reason, abrasive wear loss does not linearly increase with the applied load: in particular, an excess of PK causes lower wear under high load compared to low load due to self-lubricating properties.

### 2.6. Composition of Epoxy Coatings: Effect on Physico-Chemical Properties 

**Figure 11:**
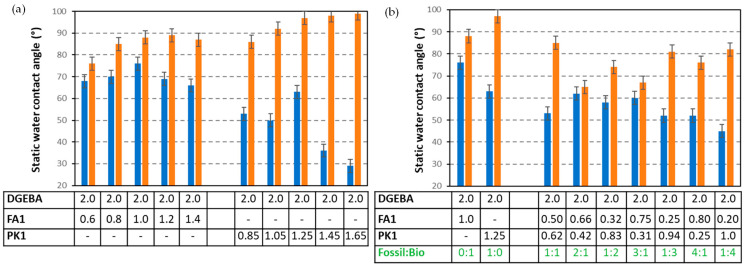
Static water contact angle of DGEBA:FA1 and DGEBA:PK1 coatings with different mixing ratios, (**a**) mixing ratio of crosslinker versus epoxy resin, (**b**) mixing ratio of fossil-based FA1 versus bio-based PK1 crosslinker. Blue bars are measured on initial coatings, orange bars are measured in the wear track, and numbers in the table are mixing ratio (g/g).

The physico-chemical surface characteristics of the epoxy coatings are determined through static water contact angles, as represented in Figure 11. The values are measured on the native coating and in the wear track for different concentrations of the FA1 and PK1 relative to the epoxy resin (Figure 11a) and for different mixtures between FA1 and PK1 (Figure 11b). For the initial coatings, the higher hydrophobicity for fossil-based versus bio-based coatings is surprisingly observed in contrast with the expectations based on the chemical structure of phenalkamines with long hydrophobic polymer chains. The predicted hydrophobicity of phenalkamines is not immediately presented for native coatings, as the hydrophobic chemical moieties are likely not favorably exposed at the surface and rather incorporated as a crosslinker in the bulk of the coatings. Indeed, the high degree of crosslinking for coatings with phenalkamines indicates the formation of a dense polymer network in the bulk of the coating. The hydrophobicity of phenalkamines becomes clear after wear damage of the coatings, as the higher water contact angles in the wear track are measured. The progressive increase in concentrations of PK1 then linearly provides the higher water contact angles, both for the coatings with PK1 (Figure 11a) or coatings with different ratios FA1:PK1 (Figure 11b).

**Figure 12:**
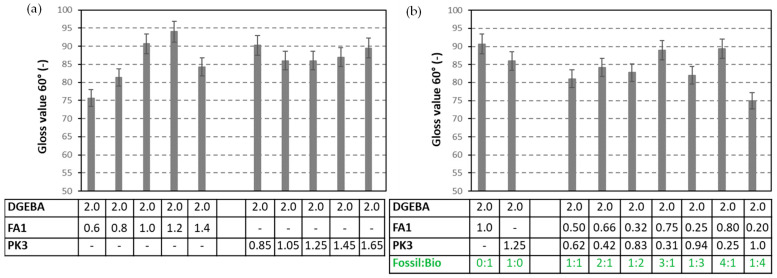
Gloss values of DGEBA:FA1 and DGEBA:PK1 coatings with different mixing ratios, (**a**) mixing ratio of crosslinker versus epoxy resin, (**b**) mixing ratio of fossil-based FA1 versus bio-based PK1 crosslinker, numbers in table are mixing ratio (g/g).

Although all coatings were clear and transparent on the wood substrates, small variations in surface gloss were measured depending on the concentrations of crosslinkers (Figure 12). The gloss for coatings with FA1 is higher compared to coatings with PK1 under a stoichiometric mixing ratio, but the variations in gloss with coating composition are smaller for the bio-based coatings. Mainly, the strong reduction in gloss for coatings with excess of free greasy amine was reported before due to the blushing of free amine present at the surface with moisture and carbon dioxide in the atmosphere [58]. The latter reaction leads to the formation of an insoluble white salt on the coating surface for fossil amines, which was presently not observed for coatings with phenalkamine, owing to the higher crosslinking density and lower tendency for migration of amine crosslinker to the surface. The trend in gloss value for different mixing ratios between fossil and bio-based crosslinkers can be explained in parallel with the homogeneity of mixing (see Supplementary Information Figure S2a), or the alkyl chain preference to the surface of phenalkamine may result in more consistent gloss for DGEBA:PK1.

### 2.7. Relation of Coating Performance with Intrinsic Mechanical Properties 

**Figure 13:**
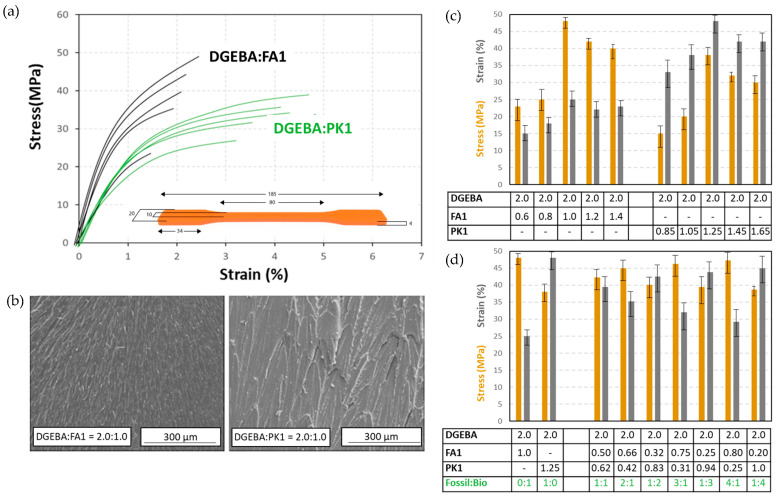
Mechanical testing of DGEBA:FA1 and DGEBA:PK1 cast films, including (**a**) illustration for some stress vs. strain curves for different mixing ratios, (**b**) scanning electron microscopy of cryogenic fracture surfaces, (**c**) summary of stress and strain data for DGEBA:FA1 and DGEBA:PK1 at different mixing ratios of crosslinker versus epoxy resin, (**d**) summary of stress and strain data for DGEBA:FA1 and DGEBA:PK1 at different mixing ratios of fossil-based FA1 versus bio-based PK1 crosslinker, numbers in the table are mixing ratio (g/g).

The results from standard mechanical testing of DGEBA:PK1 and DGEBA:FA1 cast films are presented in Figure 13, including the maximum stress at break (MPa) and strain at break (%). It can be noticed that the PK-epoxy presents higher elongation and lower strength, therefore being more ductile. The FA-epoxy presents higher strength and lower elongation and is more brittle. These findings are confirmed by microscopic observation of the cryogenic cross-section of the fracture surface for each epoxy type (represented at stoichiometric mixing ratio), differing between brittle fracture (DGEBA:FA1) and ductile fracture (DBEGA:PK1). 

**Figure 14:**
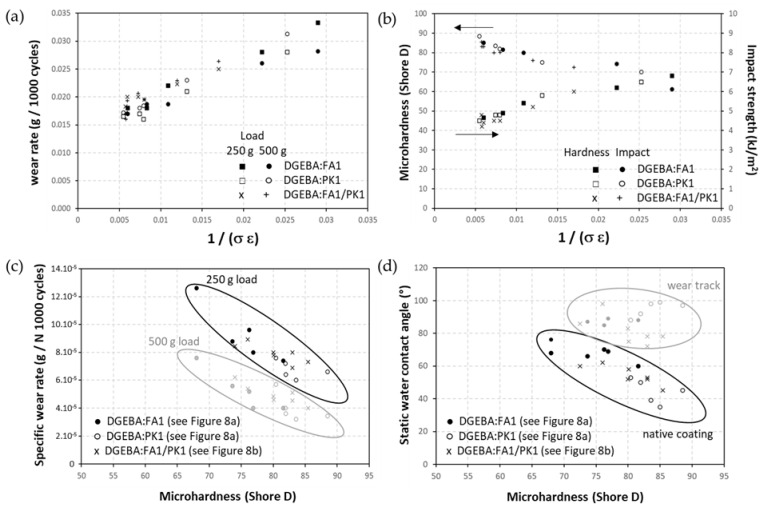
Relationship between mechanical and physico-chemical properties of DGEBA:FA1 and DGEBA:PK1 coatings with different compositions, including different mixing ratios of PK1/FA1 crosslinkers, (**a**) abrasive wear against ductility (Lancaster plot), (**b**) microhardness and impact strength against ductility, (**c**) specific wear rates versus microhardness, (**d**) static water contact angle versus microhardness.

In overall summary, the relationships between coating properties and intrinsic mechanical properties for DGEBA:FA1, DGEBA:PK1, and DGEBA:FA1/KP1 at different mixing ratios are presented in Figure 14. The unique linear correlation between abrasive wear loss and material ductility, known as the Lancaster plot (Figure 14a), applies to all coating compositions independently of the crosslinker type and illustrates that the intrinsic mechanical properties in relation to the degree of crosslinking are predominant in controlling the wear properties. The hardness and impact strength also relates to the intrinsic mechanical properties (Figure 14b), ranging from hard and brittle (FA-epoxy) to more ductile properties (PK-epoxy). The microhardness and specific wear rates (Figure 14c) also indicate singular trends for the coatings with FA1 or PK1 crosslinkers. For all relationships, the good overlap in compositions, either including the FA1, PK1, or ratio FA1/PK1 crosslinkers, confirm that coating performance can be related to intrinsic material parameters independently of fossil- or bio-based origin. As the increase in microhardness is indeed a measure of the degree of crosslinking density, it is evident that the specific wear rates decrease due to better mechanical resistance. Both for low loads and high loads abrasive wear, the single trend between the specific abrasive wear and hardness for different epoxy coating compositions confirms good compatibility of the coating compositions, including different mixing ratios of DGEBA:FA1, DGEBA:PK1 (wear data based on Figure 8a) and mixed bio-based and fossil-based crosslinkers FA1/PK1 (wear data based on Figure 8b). Additionally, the static water contact angle (Figure 14d) decreases with higher microhardness, likely due to a lower concentration of free (i.e., non-crosslinked) hydrophobic moieties at the surface for the coatings with a lower degree of crosslinking. Additionally, in the case of other bio-based acrylate coatings, the presence of free bio-based monomer fractions was responsible for high hydrophobicity [59]. Alternatively, the water contact angles in the wear track obviously do not relate anymore to the intrinsic hardness, as they depend on chemical and topographical surface modifications after wear. In conclusion, the control of intrinsic mechanical properties by the crosslinking mechanism is predominant in comparing performance between epoxy coatings with selected fossil-based and bio-based crosslinkers.

## 3. Materials and Methods

### 3.1. Chemicals and Materials

The liquid epoxy resin of bisphenol A diglycidyl ether (DGEBA) or 2,2-bis [4-(2,3-epoxypropoxy)fenyl]propane was purchased (Resion Resin Technology, Moordrecht, The Netherlands), with trade name EP101 and epoxy equivalent weight EEW = 200 g/eq. 

A range of fossil amine (FA) and bio-based phenalkamine (PK) crosslinkers was selected from commercially available grades and according to common industrial practice. All FA and PK grades were included in the first evaluation of the crosslinking kinetics, while the FA1 and PK1 grades were further used for a comparative study of coating performance. An overview of the composition and main chemical components is given in Table 1 as follows:

- The fossil amine crosslinkers include (i) FA1: a fast crosslinking fossil cycloaliphatic amine containing a mixture of 3-aminomethyl-3,5,5-trimethylcyclohexylamine (30 to 50 wt.-%) and m-phenylene bis(methylamine) (10 to 30 wt.-%) with amine hydrogen equivalent weight AHEW = 100 g/eq and trade name EP113 (Resion Resin Technology, Moordrecht, The Netherlands), (ii) FA2: a low-molecular weight amine of 1,2-ethylenediamine (EDA), with AHEW = 15 g/eq, (iii) FA3: a mixture of four low-molecular weight triethylenetetramines (TETA) with close boiling points including a linear molecule (i.e., N,N′-bis-(2-aminoethyl)-1,2-ethanediamine), a branched molecule (i.e., tris-(2-aminoethyl)amine), and two cyclic molecules (i.e., N,N′-bis-(2-aminoethyl)piperazine or bis AEP + N-[(2-aminoethyl)2-aminoethyl]piperazine) or PEEDA), with AHEW = 24 g/eq, and (iv) FA4: an amine-terminated polyoxypropylene glycol or polyetheramine (2N) with trade name Jeffamine D-230 with AHEW = 60 g/eq, supplied by Huntsman (Kortenberg, Belgium).
- The bio-based solvent-free phenalkamine crosslinkers include (i) PK1: cardamine H811 obtained as a pure cardanol-based phenalkamine (100 wt.-%) after reaction between cardanol and 1,2-ethylenediamine, with AHEW = 125 g/eq (Anacarda, Wigan WN, UK), (ii) PK2: Lite-2002 including cardanol-based phenalkamine (82 to 88 wt.-%), m-phenylene bis(methylamine) (1 to 2 wt.-%), tetraethylenepentamine (4 to 6 wt.-%) with AHEW = 104 g/eq (Cardolite, Gent, Belgium), (iii) PK3: GX-6004 including cardanol-based phenalkamine (50 to 62 wt.-%), m-phenylene bis(methylamine) (18 to 22 wt.-%), decarboxylated CNSL extract (16 to 20 wt.-%), 2,2,4-trimethylhexane-1,6-diamine (5 to 7 wt.-%) with AHEW = 76 g/eq (Cardolite, Gent, Belgium), and (iv) PK4: NX-6019 including cardanol-based phenalkamine (68 to 75 wt.-%), m-phenylene bis(methylamine) (15 to 20 wt.-%), 2,4,6-tris(dimethylamino)methyl phenol (3 to 4 wt.-%), dimethylaminopropylamine (2 to 3 wt.-%) with AHEW = 133 g/eq (Cardolite, Gent, Belgium). The compositions of commercial PK are indicative, as full details are proprietary information and cannot be disclosed. In particular, the concentration of m-phenylene bis(methylamine) as a reactive crosslinker differs in commercial grades determining the reactivity of crosslinking. In the following discussions, however, coating properties will be more importantly related to data from intrinsic thermal analysis and mechanical testing rather than an exact chemical composition.

### 3.2. Coating Formulation and Application

The coating formulations were made from DGEBA epoxy resin with FA or PK crosslinkers in various mixing ratios. Two testing series are included evaluating the effects of crosslinker types and mixing ratios:

In the first series, the stoichiometric ratio between resin and crosslinker was calculated from EEW and AHEW values in order to study the influence of crosslinker type on crosslinking behavior and kinetics. The small batches of each composition were made with the amount of crosslinker (weight *m_h_*) to be mixed stoichiometrically with 10 g of the DGEBA epoxy resin calculated from Formula (1):

(1) $$m_{h}\left(g\right)=\frac{AHEW}{EEW}\cdot 10(g)$$

In a second series, the influences of variations in mixing ratio DGEBA:FA1 or DGEBA:PK1 on coating properties were investigated by preparing concentration series with different amounts of crosslinker mixed with a given amount of 10 g DGEBA epoxy resin. For FA1, the theoretical stoichiometric ratio of 2:1 (DGEBA:FA1) was varied in experimental coating compositions with DGEBA:FA1 ratios between 2:0.3 to 2:1.4 by increasing the amount of crosslinker (i.e., 3, 4, 5, 6, or 7 g FA1 added to 10 g DGEBA, respectively). For the PK1, the theoretical stoichiometric ratio of 2:1.25 (DGEBA:PK1) was experimentally varied between 2:0.85 to 2:1.65 by increasing the amount of crosslinker (i.e., 4.25, 5.25, 6.25, 7.25, and 8.25 g PK1 added to 10 g DGABA, respectively). In addition, the compatibility of both crosslinkers and the progressive replacement of fossil-based FA1 into bio-based PK1 was investigated by changing the PK1:FA1 ratio between 1:1, 2:1, 1:2; 3:1, 1:3, 4:1, and 1:4, relatively to 10 g DBEBA, respectively. 

The coatings were manually applied by a doctor blading onto 10 × 10 cm^2^ wood samples (softwood beech) that were surface planed and overnight dried in a circulating air oven at 60 °C. The wet film thickness of the coatings was constant at 70 µm as determined through the application blade. The dry coating thickness was around 68 ± 3 µm for all samples and close to the applied blade thickness, as coatings do not contain solvent. The thermal curing conditions were varied by including a precure at a given temperature of 60 °C for 0, 1, 6, and 12 h, followed by one-day storage under controlled laboratory conditions (25 °C, 60% RH). The coatings were further cured for one month under controlled laboratory conditions (25 °C, 60% RH) without thermal precuring, as usual in wood coating practice. With similar compositions of the coatings, films were cast with a controlled thickness of 0.5 mm for the determination of intrinsic mechanical properties. 

### 3.3. Characterization Methods

The crosslinking of epoxy resins was studied through differential scanning calorimetry (DSC) using DSC 3+ equipment (Mettler Toledo, Columbus, OH, USA). For crosslinking of DGEBA with respective amines, a single non-isothermal run was completed on a liquid sample mass of 4 mg heated in a hermetically sealed aluminum pan between −50 to 200 °C at 5 °C/min under nitrogen atmosphere. An exothermal peak in the heat flow curve was recorded as crosslinking happened, from which the degree of crosslinking (%) and conversion rate (dα/dt) were calculated. Similarly, the solid epoxy coatings were analyzed on a sample mass of 7 mg during two heating cycles between −50 to 200 °C at a rate of 10 °C/min, with an intermediate cooling step at a similar rate. The residual exothermal peak in the first heating cycle was used to calculate the degree of crosslinking of the respective coatings relative to a non-cured and fully cured coating, while the glass transition temperature (*T_g_*) of the fully crosslinked coating was determined from the second heating cycle. The viscosity of liquid coating formulations was determined with a low shear rotational viscosity meter and appropriately selected spindle according to ASTM D2196 (DV-III Ultra, Brookfield Engineering, Hadamar-Steinbach, Germany), applying a constant shear rate of 100 rpm over a time up to 60 min under room temperature. 

The abrasion resistance of epoxy coatings was determined on a circular taber tester with a dual rotary platform (Model 5135, Taber Industries, New York, NY, USA) following ASTM D4060-10 standard under rotational speed of 72 r/minutes and CS-10 Calibrase abrasive wheels under a load of 250 or 500 g. The abrasive wheels were reconditioned against a S-11 resurfacing disc after each full test of 1000 cycles. The abrasive wear was determined as the weight loss after 250, 500, 750, and 1000 cycles, using an analytical balance with accuracy 0.0001 g (Sartorius, Göttingen, Germany). Microhardness measurements were completed with a handheld Shore D durometer (micro hardness tester) according to ASTM D2240, using a hardened steel tip with a 30 ± 0.5° conical point and 0.100 ± 0.012 mm tip radius. Water absorption was measured by means of a Cobb ring test for according to ASTM D 5795-95 for a contact time of 2 h. The specular gloss was measured with a micro-triglossmeter (BYK-Gardner Instruments, Geretsried, Germany) under 60° following ISO 2813. The static contact angles with deionized water were measured on an OCA 50 goniometer (Dataphysics Instruments GmbH, Filderstadt, Germany), using droplets of 3 µL that were fitted by the Laplace-Young procedure in agreement with ISO 19403-2. The micro-scratching was completed with a sclerometer type 3092 (Elcometer, Aalen, Germany) having a tungsten carbide tip of 0.75 mm radius and loaded under 10 or 20 N by exchange of an appropriate spring constant, following ISO 4586-2. The observations by optical microscopy were made on a stereomicroscope MZ12 (Leica, Wetzlar, Germany) at magnifications 20× or 50×. Topographical images were obtained on a VK-X3000 laser interferometer (Keyence, Mechelen, Belgium) at magnifications of 50× or 150×. All physico-chemical and mechanical analysis was completed at 10 independent locations distributed over the coating surface and reported as average values with standard deviation.

The intrinsic mechanical properties of PK-epoxy and FA-epoxy films were determined on a universal testing machine (ProLine Z005, Zwick Roll, Haan, Germany), on standardized dog-bone tensile bars. The impact properties were determined on an Izod impact testing device measuring absorbed energy on standard sample sizes according to ASTM D256. All mechanical results are reported as an average of 10 tests per composition, including maximum stress at break (MPa), strain at break (%), and impact strength (kJ/m^2^). A scanning electron microscopy image of cryogenic fractured surfaces was taken on the TM3000 (Hitachi, Krefeld, Germany).

## 4. Conclusions

The bio-based phenalkamine (PK) crosslinkers offer benefits in curing reaction and performance of epoxy coatings relatively to a selection of fossil-based amines (FA), besides the environmental need for replacement of fossil-based amines into renewable alternatives. Although the direct one-by-one analogues in chemical structure for fossil-based and bio-based crosslinkers cannot be found, relationships could be drawn according to the intrinsic properties: overall good correspondence between crosslinking density, resulting mechanical properties and coating performance could be explained by intrinsic features of bio-based and fossil-based components.

A study on crosslinking of epoxy with selected PK indicates that the rection kinetics and viscosity are in similar range to selected fossil amines with minor differences indicating a somewhat faster crosslinking at room temperature conditions. The reduction in exothermal enthalpy for crosslinking of epoxy with PK is lower which may favor better control on the heat released during crosslinking. The residual exothermal peak after reaction of epoxy with PK crosslinkers has lower intensity, in parallel with the faster reaction kinetics and higher degree of crosslinking for PK at room temperature.

The mechanical properties of epoxy/PK coatings present higher hardness, better scratch resistance and lower abrasive wear under optimized processing conditions, in parallel with lower sensitivity of the performance to the resin/crosslinker mixing ratio compared to epoxy/FA coatings. After testing different mixing ratios between fossil-based and bio-based crosslinkers, the progressive increment in bio-based crosslinker content how linear improvement in mechanical properties indicating their compatibility. The mechanical properties can be explained in relation between the microhardness, abrasion resistance, and degree of crosslinking; in particular, the abrasive wear, microhardness and impact strength linearly relate to the ductility parameters for both epoxy/PK and epoxy/FA coatings. Indeed, only superficial surface scratches are observed after abrasive wear for the epoxy/PK coatings in contrast with wear of the coating bulk for tradition epoxy/amine coatings. In addition, the higher hydrophobicity of epoxy/PK coatings on worn surfaces ensures better protection as expected by presence of long alkyl chains in the PK polymer structure. These results may be promising for the industrial application of bio-based PK crosslinkers in industrial protective coatings on wood (e.g., flooring), where superior performance is illustrated after the optimization of processing conditions. 

## Figures and Tables

**Table 1:** Overview of composition and main components of epoxy resin (DGEBA), fossil-based crosslinkers (FA1 to FA4), and bio-based crosslinkers (PK1 to PK4).

Component	Main Chemical Ingredient
Epoxy resin DGEBA	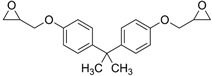
Fossil crosslinker FA1	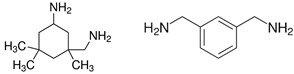
Fossil crosslinker FA2	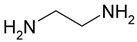
Fossil crosslinker FA3	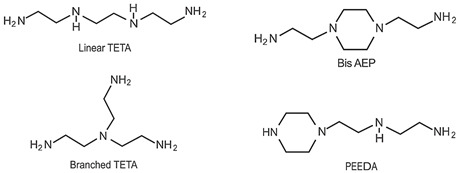
Fossil crosslinker FA4	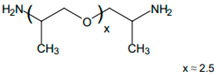
Cardanol-based Phenalkamine crosslinker PK1 to PK4	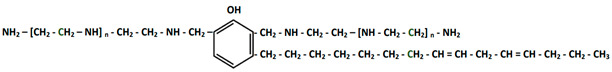 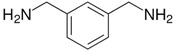

## Data Availability

The data presented in this study are available on request from the corresponding author.

## Supplementary Materials

The following supporting information can be downloaded at: https://www.mdpi.com/article/10.3390/molecules28114259/s1, Figure S1: Online measurement of abrasive wear loss as a function of the number of abrasive cycles, Figure S2: Relationships between mixing ratio of bio-based (PK1) versus fossil-based (FA1) crosslinker and coating performance, (a) microhardness, (b) gloss value; Figure S3: Online measurement of abrasive wear loss as a function of the number of abrasive cycles during Taber abrasive testing, Figure S4: Mechanical scratching tests on epoxy coatings with different ratio of FA1:PK1 crosslinkers, scratched under 10 N and 20 N normal load, Figure S5: Evaluation of abrasive wear track of DGEBA/FA1 and DGEBA/PK1 coatings with different conditions of thermal precuring, observed through laser scanning microscopy at 50× magnification.
