# Supplementary material for: Benchmark Study of Epoxy Coatings with Selection of Bio-Based Phenalkamine versus Fossil-Based Amine Crosslinkers
Source: Molecules. 2023 May 23;28(11):4259. doi: 10.3390/molecules28114259 (PMC10254157; doi:10.3390/molecules28114259)

BENCHMARK STUDY OF EPOXY COATINGS WITH BIO-BASED PHENALKAMINE VERSUS FOSSIL AMINE CROSSLINKERS

Pieter Samyn *, Joey Bosmans, Patrick Cosemans

SIRRIS, Department of Innovations in Circular Economy and Renewable Materials,

3001 Leuven, Belgium

*Correspondence: pieter.samyn@outlook.be

ORCID: 0000-0002-2180-7925

Supplementary information

Supplementary Information Figure S1.

On-line measurement of abrasive wear loss as a function of the number of abrasive cycles during Taber abrasive testing, for epoxy coatings with fossil-based (FA1, closed symbols, full lines) and bio-based (PK1, open symbols, dotted lines) crosslinker after different conditions of thermal pre-curing, (a) abrasion under low loads 250 g for coatings with 1h, 6h, 12h thermal precuring at 60°C, (b) abrasion under high loads 500 g for coatings with 1h, 6h, 12h thermal precuring at 60°C, (c) abrasion under low loads 250 g for coatings with 1h, 6h, 12h thermal precuring at 60°C and one week curing under environmental lab conditions.

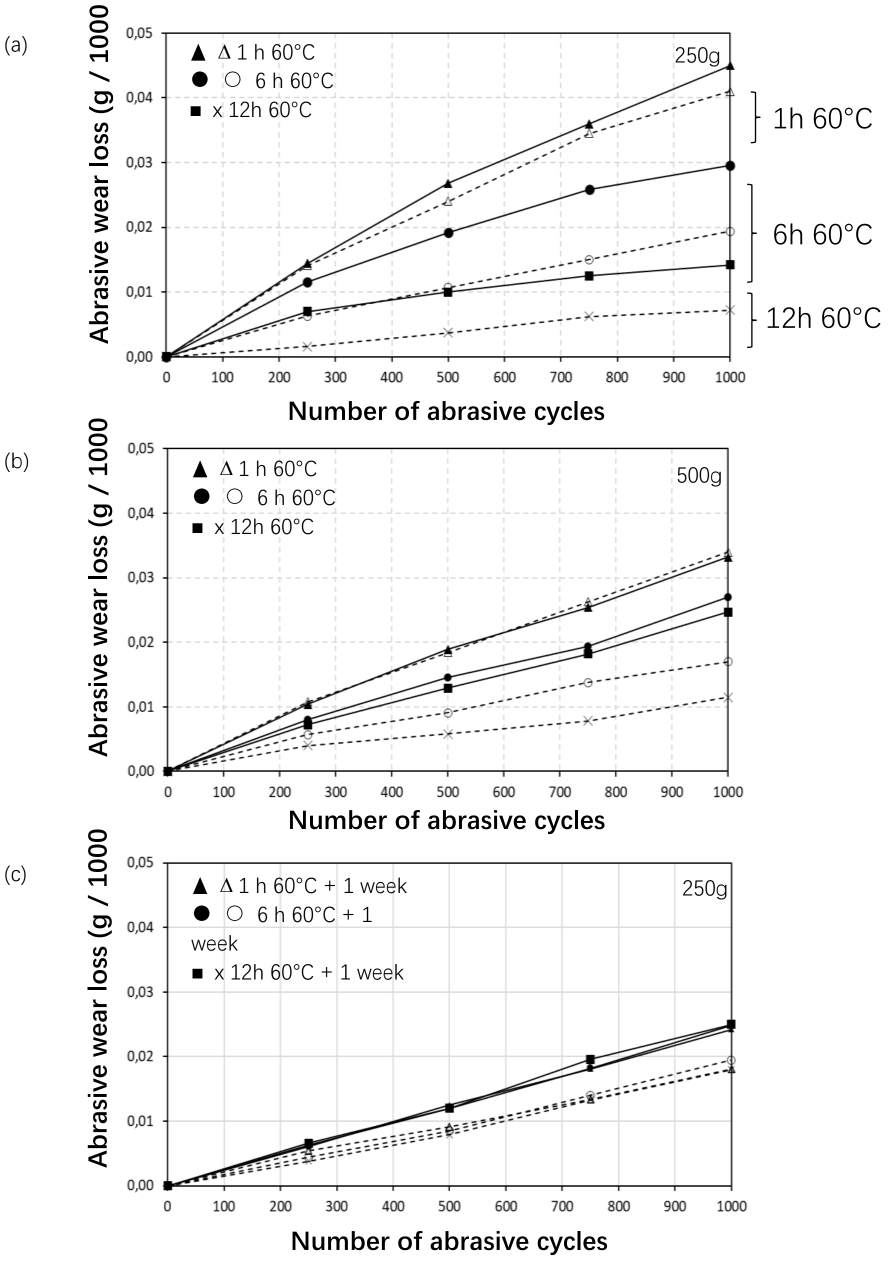

Supplementary Information Figure S2.

Relationships between mixing ratio of bio-based (PK1) versus fossil-based (FA1) crosslinker and coating performance, (a) microhardness, (b) gloss value

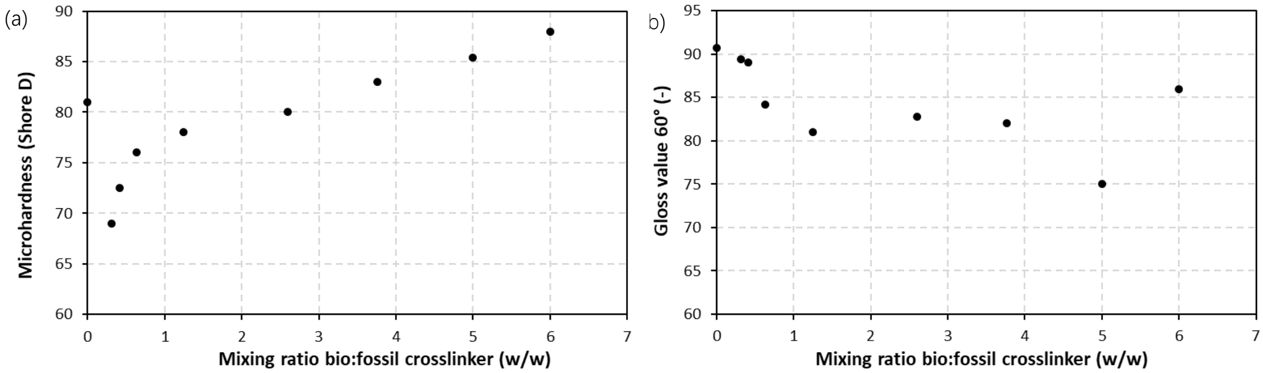

Supplementary Information Figure S3.

On-line measurement of abrasive wear loss as a function of the number of abrasive cycles during Taber abrasive testing, for epoxy coatings with fossil-based (FA1, closed symbols, full lines) and bio-based (PK1, open symbols, dotted lines) crosslinker, with different ratio of epoxy resin versus crosslinker, (a) abrasive wear under low loads (250 g), (b) abrasive wear under high loads (500 g).

**DGEBA**	2.0	2.0	2.0	2.0	2.0		2.0	2.0	2.0	2.0	2.0
**Fossil amine**	0.6	0.8	1.0	1.2	1.4		-	-	-	-	-
**PK3**	-	-	-	-	-		0.85	1.05	1.25	1.45	1.65
**Symbol**	■	●	▲	♦	X		□	○	Δ	◊	X

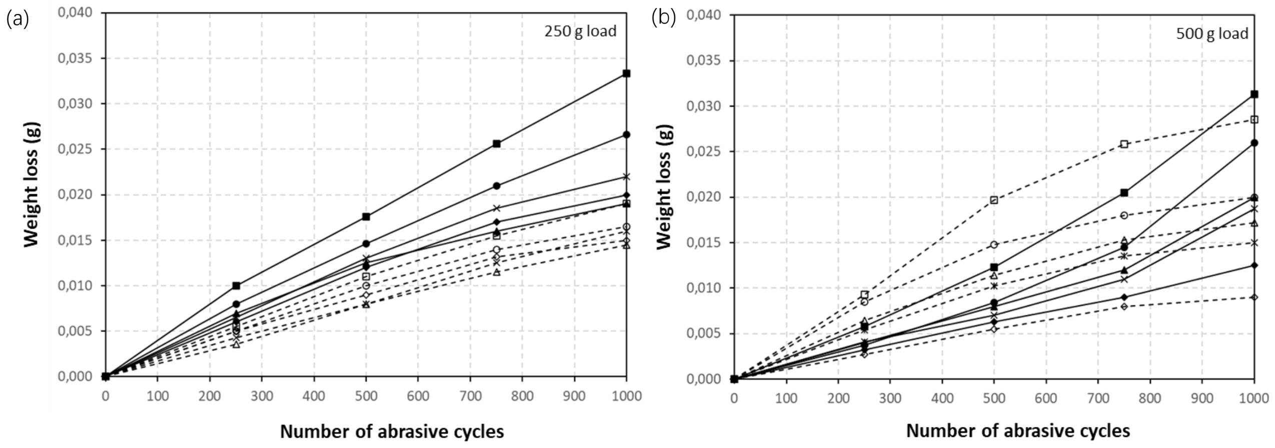

Supplementary Information Figure S4.

Mechanical scratching tests on epoxy coatings with different ratio of FA1:PK1 crosslinkers, scratched under 10 N and 20 N normal load and evaluated through optical microscopy under 20x and 50x magnification.

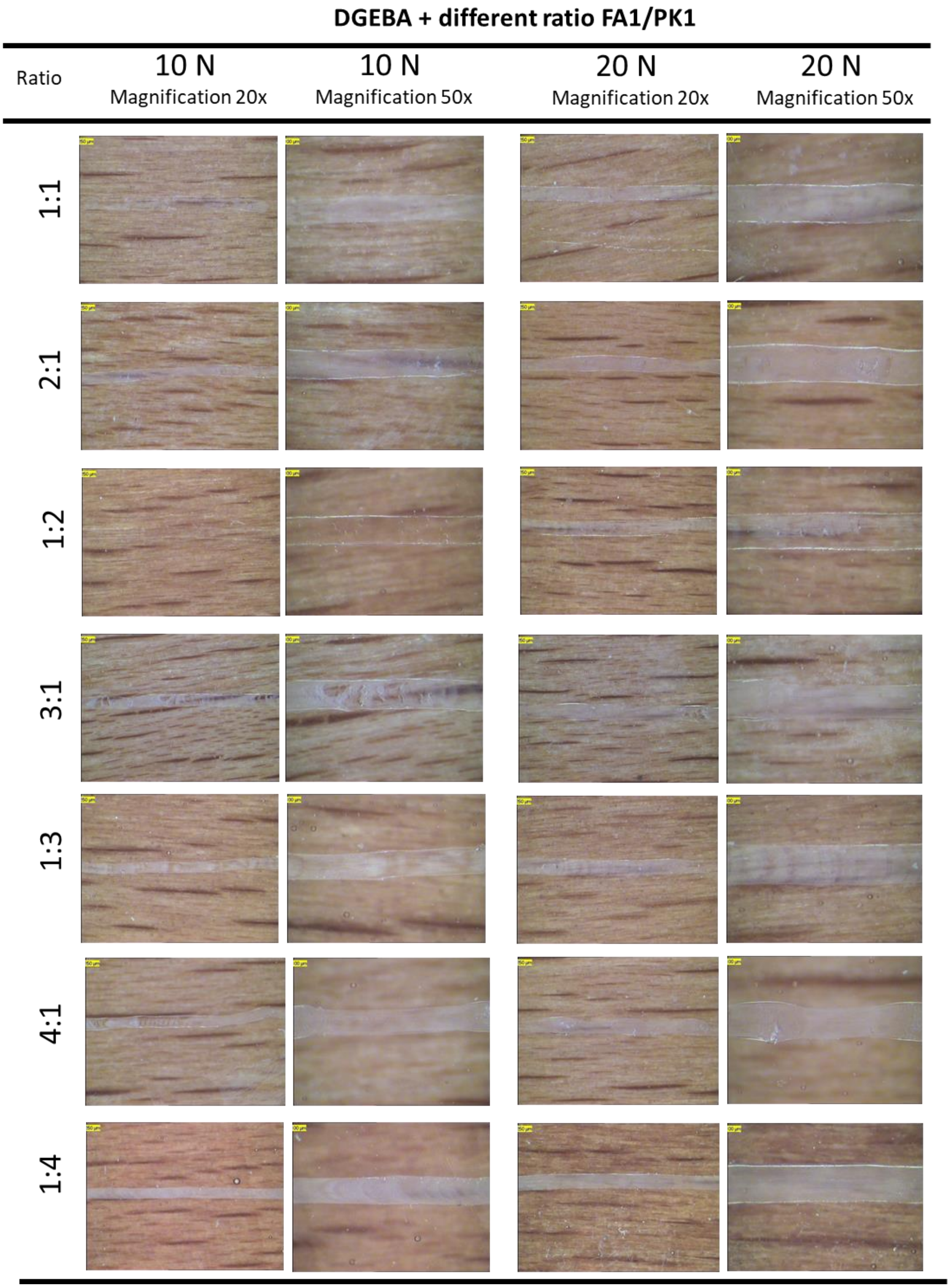

Supplementary Information Figure S5.

Evaluation of abrasive wear track of DGEBA/FA1 and DGEBA/PK1 coatings with different conditions of thermal precuring, observed through laser scanning microscopy at 50x magnification.

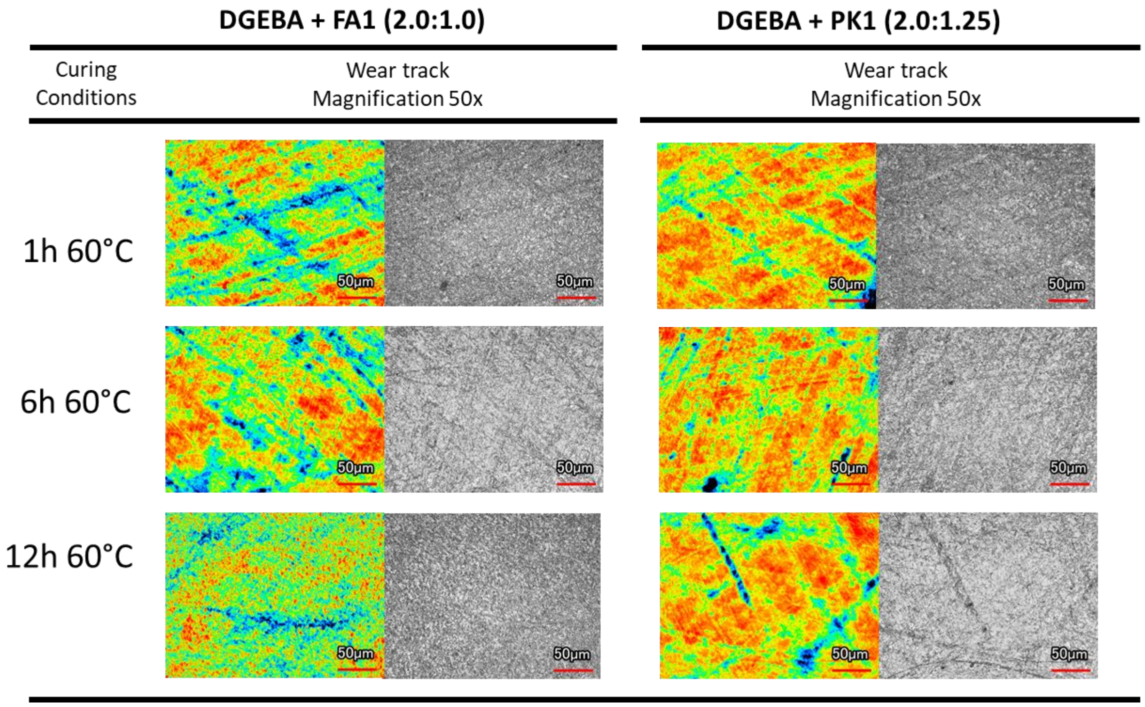

Supplement: Supplementary file 1 [file molecules-28-04259-s001.zip › molecules-2402859-supplementary.pdf]
